# Intraoperative Flow Cytometry for the Rapid Diagnosis and Validation of Surgical Clearance of Non-Melanoma Skin Cancer: A Prospective Clinical Feasibility Study

**DOI:** 10.3390/cancers16040682

**Published:** 2024-02-06

**Authors:** Georgios Markopoulos, Evangeli Lampri, Ioulia Tragani, Nikolaos Kourkoumelis, Georgios Vartholomatos, Konstantinos Seretis

**Affiliations:** 1 Haematology Laboratory-Unit of Molecular Biology and Translational Flow Cytometry, University Hospital of Ioannina, 45110 Ioannina, Greece; geomarkop@gmail.com (G.M.); gvarthol@gmail.com (G.V.); 2 Department of Pathology, Medical School, University of Ioannina, 45110 Ioannina, Greece; elampri@uoi.gr (E.L.); itragani@hotmail.com (I.T.); 3 Department of Medical Physics, Medical School, University of Ioannina, 45110 Ioannina, Greece; nkourkou@uoi.gr; 4 Department of Plastic Surgery, Medical School, University of Ioannina, 45110 Ioannina, Greece

**Keywords:** non-melanoma, skin cancer, diagnosis, flow cytometry, intraoperative flow cytometry, translational oncology

## Abstract

**Simple Summary:**

There is escalating incidence of non-melanoma skin cancer (NMSC), which is the most prevalent cancer in humans. Our current research introduces intraoperative flow cytometry (iFC) as a diagnostic tool for NMSC and its surgical margins. Flow cytometry is instrumental in measuring and quantifying cell attributes. We conducted a pilot study involving patients diagnosed with NMSC. Our findings underscore the potential of iFC as an invaluable tool in characterizing NMSC and ensuring tumor-free resection margins. Given the technique’s proven efficacy in other cancer types, we believe iFC offers a promising avenue for enhancing complete tumor excision rates in NMSC treatment.

**Abstract:**

Non-melanoma skin cancer (NMSC) is the most prevalent cancer in humans, with a high global incidence. We present a prospective clinical feasibility study on the use of intraoperative flow cytometry (iFC) for the instant diagnosis of NMSC and its complete surgical clearance. Flow cytometry, a laser-based technique, quantifies cell features, which has applications in cancer research. This study aim is to explore the potential applicability of iFC in detecting and characterizing NMSC and its surgical margins. In total, 30 patients who underwent diagnosis for NMSC were recruited. The method demonstrated high sensitivity (95.2%) and specificity (87.1%), with an accuracy of 91.1%, as confirmed with a receiver operating characteristic curve analysis. The results also indicated that most tumors were diploid, with two cases being hypoploid. The average G0/G1 fractions for normal and tumor tissue samples were 96.03 ± 0.30% and 88.03 ± 1.29%, respectively, with the tumor index escalating from 3.89 ± 0.30% to 11.95 ± 1.29% in cancerous cells. These findings underscore iFC’s capability for precise intraoperative NMSC characterization and margin evaluation, promising enhanced complete tumor excision rates. Given the technique’s successful application in various other malignancies, its implementation in NMSC diagnosis and treatment holds significant promise and warrants further research in clinical trials.

## 1. Introduction

Non-melanoma skin cancer (NMSC) presents the most frequent malignancy in humans, demonstrating a consistent and progressively increasing annual incidence rate [1,2,3]. In the United States, the occurrence of NMSC surpasses the combined incidence of all other types of cancer [1]. Similarly, data from the GLOBOCAN 2020 estimates of cancer incidence and mortality predict a 5-year prevalence of almost 6.5 million cases worldwide [4]. The vast majority of NMSCs, also referred to as keratinocyte cancers, are attributed to basal cell carcinoma (BCC) and squamous cell carcinoma (SCC). Although in the pathogenesis of these cancers several mechanisms are involved, ultraviolet radiation (UVR) constitutes the primary factor leading to DNA damage in keratinocytes [5,6]. In fact, the accumulated amounts of ultraviolet radiation, along with patient’s Fitzpatrick type and age, immunosuppression, and prior radiotherapy are the most common risk factors for NMSC development [7,8,9,10].

Although the mortality associated with these tumors is relatively low, they impose a substantial economic burden on healthcare systems globally, primarily due to their high prevalence [11]. Moreover, they are linked to significant morbidity, particularly in patients with facial tumors that cause extensive local damage, often necessitating challenging reconstructive operations [12].

The treatment of NMSC remains mostly surgical, in accordance with the relevant international guidelines [13,14,15]. The adequate tumor margin resection is not universally accepted, as it is influenced by several variables defined in these guidelines and other clinical studies [13,15,16]. The clinical heterogeneity of NMSC, the difficulty to accurately determine the clinical tumor boundaries, and the constant effort to manage these common tumors in a logical and adequate manner was the foundation of this proof-of-concept study through implementing intraoperative flow cytometry (iFC).

Flow cytometry stands as the benchmark laser-driven method for assessing and quantifying cellular characteristics [17,18]. It serves as an essential tool in foundational, translational, and clinical investigations, offering numerous uses in cancer research such as cancer cell immunophenotyping, detailing hematological cancers, identifying measurable leftover disease and metastatic shifts, and evaluating DNA quantity for cell cycle analysis and ploidy assessment [19,20]. The efficacy of cytometry hinges on the employment of a flow cytometer, specific agents like fluorochromes, for instance, fluorescein, and specialized data interpretation software [18]. This method offers a swift and precise data gathering approach, ensuring speedy cellular evaluations with consistent results. Sources for samples encompass formalin-preserved cells, cryopreserved specimens, fresh tissues, and paraffin-encased tissues. The sole essential condition is properly executed pre-processing, ensuring cells are dispersed in a consistent single-cell mix [14]. The beneficial attributes of this method have paved its way for application as an on-the-spot diagnostic instrument in brain cancer surgeries and, subsequently, various other cancers, emerging currently as an innovative adjunctive tool for the diagnosis and treatment of cancer [21]. Apart from brain cancer analysis, iFC has been successfully used in cancer and has verified its utility in a number of malignancies, including head and neck tumors, gynecological, breast, bladder, and gastrointestinal tract cancers [22,23,24,25,26,27].

The aim of this study was to implement a rapid intraoperative flow cytometry process, and investigate its value for the characterization of NMSC and tumor-free resection margins. We present a pilot study of analysis of NMSC cases and its relevant margin tissues in which, the feasibility and the accuracy of the methodology to define skin cancer tissue was examined.

## 2. Materials and Methods

The sample of the current study included patients with a non-melanoma skin cancer diagnosis, treated surgically with K.S. in the Department of Plastic and Reconstructive surgery at the University Hospital of Ioannina, a tertiary hospital of the region of Epirus. The study protocol has been approved by the Research Committee of the University Hospital of Ioannina (with approval number: 2/31-1-2023[θ.15]), and has been conducted in accordance with the principles presented in the Declaration of Helsinki.

### 2.1. Tumor Sample Collection

During surgery, the tumor was completely removed in a regular manner, oriented with stitches by the surgeon (K.S.), and placed on a sterile surface. Cancer cells were collected with a scalpel from the specimen, avoiding areas of macroscopical necrosis. A total of three to five samples were taken: one from the tumor itself and two to four from the peripheral margins. A 1 mm width of tissue was removed from the four peripheral margins, and the deep margin of the macroscopically healthy wound of the patient. This method was partially based on previous iFC methods [23,26]. The tumor specimen was always sent to an expert pathologist (E.L.) to conduct frozen-section pathology evaluation, which remains the gold standard for tumor diagnosis and margins assessment. Both the pathology evaluation and analysis with flow cytometry were blinded.

### 2.2. DNA Content Analysis (Ioannina Protocol)

DNA analyses were performed immediately after tumor extirpation, using the Ioannina protocol, which was initially developed for brain tumor characterization [21]. Briefly, cells were uniformly mixed for 1 min in phosphate-buffered saline, then sieved to eliminate clumps and achieve single-cell mixtures. An automated hematology device was used to count the cells, resulting in a concentration of 10^6^ cells/mL. Immediate processing for staining was completed by introducing propidium iodide (125 μg/mL) in alive, unfixed cells, using Ioannina Protocol staining solution (for details see ref. [21]). After a 3 min interval, the cells underwent flow cytometric examination using a flow cytometer (FACSCalibur by BD Bioscience, Franklin Lakes, NJ, USA), and the analysis was performed using CellQuest V3.1 software (BD Bioscience). Standard peripheral blood mononuclear cells, sourced from healthy contributors via a Ficoll gradient (Ficoll-Paque division, GE Healthcare, Little Chalfont, Buckinghamshire, UK), have been employed for calibrating the flow cytometer and pinpointing the G0/G1 peak of diploid cells in DNA charts. Each sample had around 5000 events (cell nuclei) assessed. A gating approach, focusing on the area/width evaluation of propidium iodide glow, was applied to exclude cell pairs from DNA content measurement (Figure S1), as mentioned earlier [22]. Each sample took about 5–6 min for a complete analysis.

From this evaluation, two metrics were derived: Firstly, a DNA index, the quotient of the geometric mean fluorescence of the G0/G1 peak of tumor cells to that of standard cells. A tumor with a DNA index surpassing 1.1 was deemed aneuploid/hyperploid, whereas one with a DNA index less than 0.9 was labeled aneuploid/hypoploid. If the DNA index was determined to be 1, the tumor was categorized as diploid. Second, a tumor index, the cumulative fraction of cells in both S and G2/M cell cycle phases, was also calculated. The tumor index indicates cell proliferation potential, which is a hallmark of cancer [28].

### 2.3. Statistical Analysis

We employed the Mann–Whitney U test to contrast the cell cycle phase fractions of cancer with the respective ones of normal cells. Continuous figures were presented as the average ± standard variation. Data in the graphs are depicted as medians and the measure of data scatter in the graphs is the interquartile range. To ascertain the best threshold values and compute the method’s sensitivity and specificity (sensitivity refers to iFC’s ability to correctly identify tumors, and specificity is its ability to correctly identify non-tumor tissue), we utilized the receiver operating characteristic (ROC) curve evaluation to detect cancer cells in a given sample. A probability value less than 0.05 has been considered significant. For all analyses, an SPSS statistical software package, version 23 (IBM, New York, NY, USA) was used, and then viewed with GraphPad software version 8.4.2.

## 3. Results

Our proof-of-concept study included 30 patients aged 43–91 years (mean age: 78 years) who underwent surgery for non-melanoma skin cancers treatment. The skin tumor sample as well as the respective margin samples were collected, and real-time intraoperative flow cytometry was performed to determine tumor proliferation, and evaluate the resection margins. Individual patients’ characteristics—which included age, the results of flow cytometry analysis, and the final pathology assessment report—are presented in Table 1.

**Figure 1:**
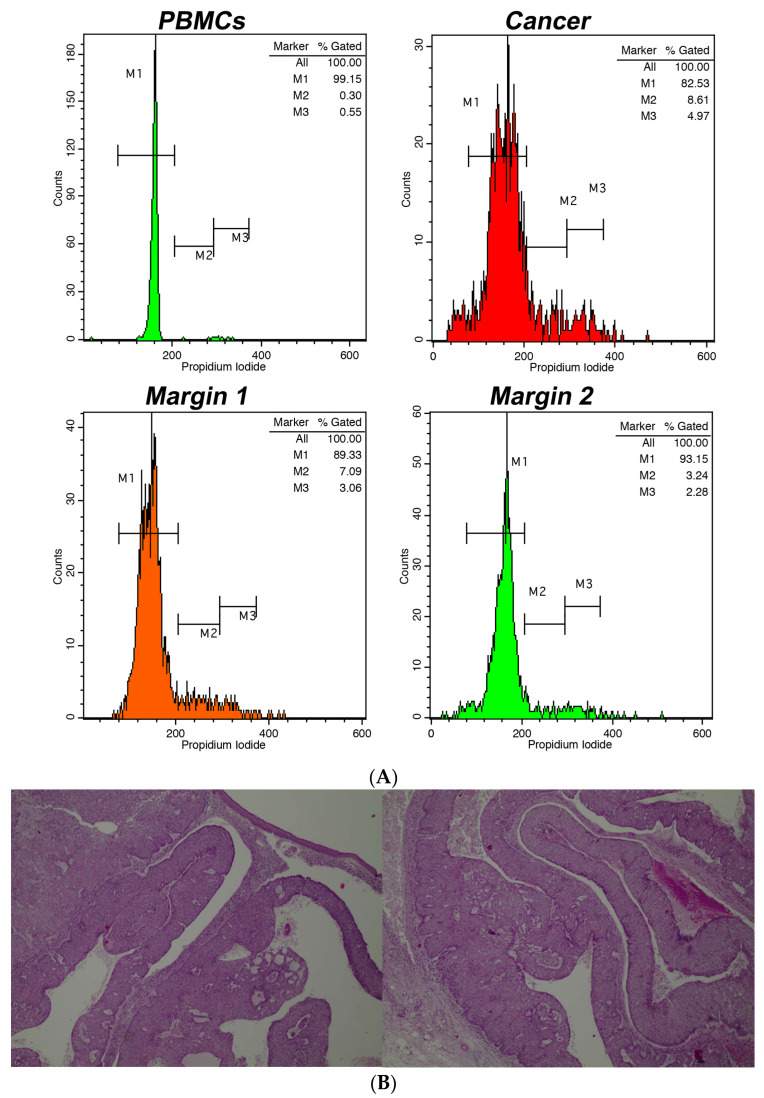
(**A**). Intraoperative flow cytometry for DNA assessment. The markers M1, M2, and M3 denote the cell fractions in G0/G1, S, and G2/M phases, respectively. Top left chart: spread of peripheral blood mononuclear cells/PBMCs (shown in green); Top right chart: spread of cancerous cells (depicted in red). The showcased instance is diploid, with a DNA index of 1 and a tumor index around 18%. Bottom left chart: a tumor edge (in orange) with a tumor index approximately 10.5%; Bottom right chart: a tumor boundary (in green) with a tumor- index close to 5.5%. (**B**). Partial view in two photos of the whole depth (5 mm) of a corresponding BCC, nodular type. Hematoxylin & eosin. Magnification, ×40.

Our study provides significant insights into the application of intraoperative flow cytometry (iFC) for real-time DNA assessment during non-melanoma skin cancer (NMSC) surgeries. A representative analysis, along with the corresponding pathological examination, is presented in Figure 1, which showcases the utilization of iFC in distinguishing between different cell types and their respective phases. The assessment of DNA content is highlighted, with an example of a diploid tumor exhibiting a DNA index of 1 and a tumor index of 18%. Furthermore, a detailed exploration at the microscopic level is presented in Figure 1B. The histological examination confirms the presence of cancerous cells and reveals the structural nuances of a nodular type Basal Cell Carcinoma (BCC) to the depth of 5 mm, substantiating the findings of the iFC analysis.

First, the DNA index was calculated. In most cases, the tumors were diploid. Two cases were hypoploid, containing a DNA index of 0.8. The results of ploidy assessment, based on DNA index calculation are presented in Table 1.

**Figure 2:**
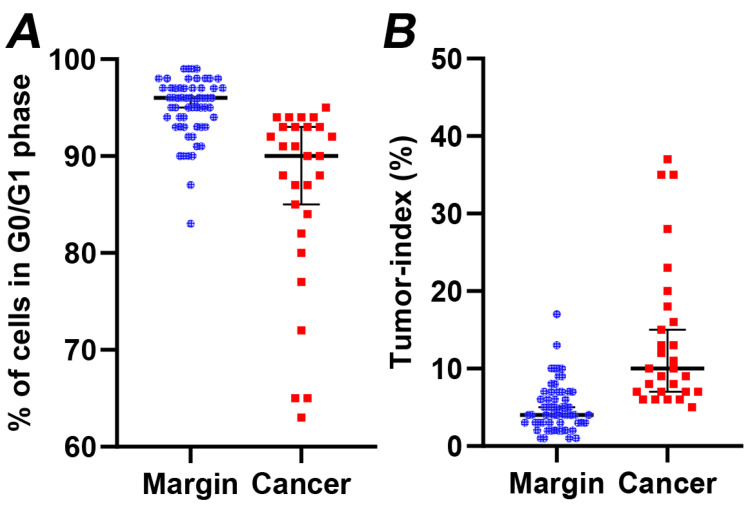
(**A**) The percentage of cells in G0/G1 phase and (**B**) the tumor index (cumulative percentage of cells in S and G2/M cell cycle phases) in margin (denoted with blue dots) versus cancer samples (denoted with red dots), calculated following iFC analysis. Median percentages are shown as horizontal lines in each group. There is a significant difference between the two groups (*p* < 0.005).

**Figure 3:**
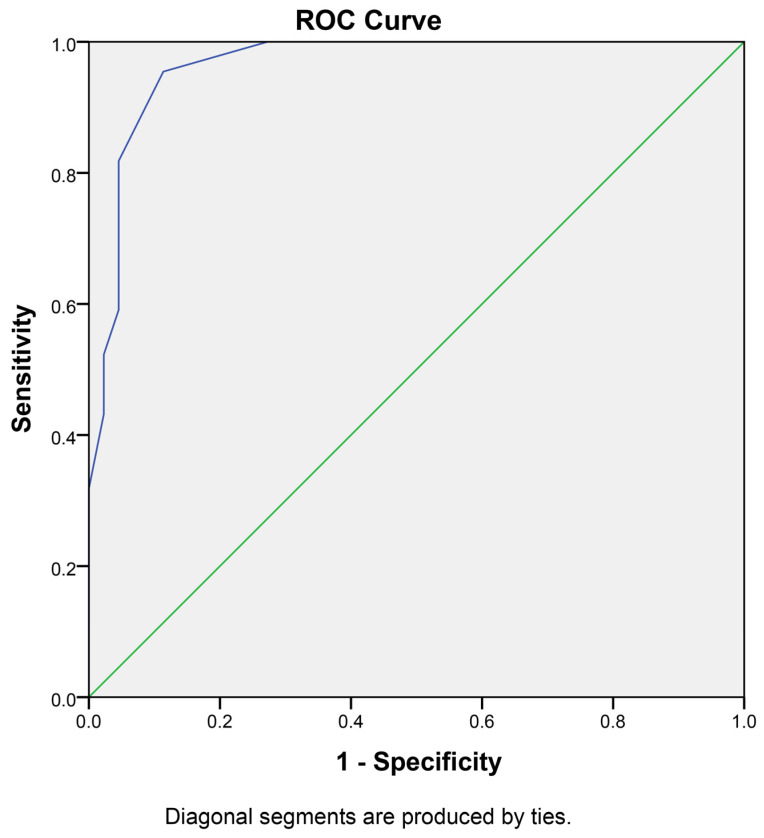
Receiver operating characteristic (ROC) curve for iFC in NMSC diagnosis: This ROC curve illustrates the performance of intraoperative flow cytometry (iFC) in distinguishing between normal and cancerous tissue samples in NMSC. The analysis includes 46 negative samples (2 AK and 44 negative margins) and 42 cancer samples (28 primary cancer samples and 14 positive margins), characterized using iFC and verified by an experienced pathologist. The area under the curve (AUC) is 0.947 with a standard error of 0.023 (*p* < 0.001).

Subsequently, we examined the cell distribution across different cell cycle stages. The average G0/G1 fractions for normal and tumor tissue samples stood at 96.03 ± 0.30% and 88.03 ± 1.29%, respectively (as seen in Figure 2A). The G0/G1 phase fraction in margins was notably greater (*p* < 0.005) compared to that in cancer cells. Conversely, the average tumor index increased significantly from 3.89 ± 0.30% to 11.95 ± 1.29% (illustrated in Figure 2B). The peak tumor index was determined to be 37%, with a median value of 9%. Overall, the growth capacity of the cancer cells is reflected in a marked increase in the tumor index, as depicted in Figure 3. The total results of iFC analysis are presented in Table S1.

In order to calculate the sensitivity and specificity of the current assay and the optimal cutoff value for the differentiation between a normal from a cancerous sample, a receiver operating characteristic (ROC) curve analysis was performed. To this end, the negative samples (46 in total) were considered normal tissue, and were analyzed against the 28 cancer samples along with 14 positive margins (42 in total). The margin status has been verified following pathology assessment by an experienced pathologist and the classification was based on the verified normal versus verified cancerous tissue. In total, using pathology as the gold-standard method, the assay had a high specificity, with one false-positive and two false-negative results. We should mention that the skewed ratio in the positive sample pool is a result of that fact that the representation was limited by the available sample numbers. The optimal cut-off value to delineate whether a given tissue contains cancer cells is a tumor index of more than 5.75% (or a G0/G1 percentage of less than 94.25%), resulting in 95.2% sensitivity and 87.0% specificity, introducing an accuracy to our assay of 91.1% (Figure 3 and Table S2), indicating a significant differentiation capability of the iFC method.

## 4. Discussion

Flow cytometry has demonstrated its efficacy as a potent method for the rapid and comprehensive analysis of heterogeneous cell populations, especially in the discipline of hematology, providing an increased detection capability at the single-cell level [29]. The distinctive features of this method can be effectively implemented in tumor diagnostics, enabling the monitoring of clonal expansion in cancer cells as subpopulations exhibiting distinct genetic characteristics [30,31]. In addition, iFC enables accurate intraoperative characterization of the tumor margins, providing improved complete tumor excision rates, as the evidence from its application in different surgical disciplines has recently shown. During the last decade, iFC has been successfully applied in brain, head-and-neck, gastrointestinal tract, and gynecological malignancies [22,23,24,25,26,27]. To the best of our knowledge, this proof-of-concept study is the first clinical application of iFC in the diagnostic assessment of NMSC and its margins.

Hanahan has recently outlined the eight core hallmarks of cancer as an integrative concept to enable comprehensive understanding of the mechanisms underlying cancer development and progression, and we utilize this knowledge in the field of cancer precision medicine [28]. The genomic instability and induction of mutations, the induced and sustained proliferative signaling, as well as the capability for evading growth suppressors are prevalent features of cancer, and thus changes in chromosome quantity and structure are anticipated. The chromosome instability is correlated with aneuploidy, resulting in ongoing karyotypic changes that contribute to tumor heterogeneity, resistance to drugs, and eventual treatment failure [32]. Yan et al. reviewed the clinical applications of aneuploidies as prognostic biomarkers in the evolution of lung cancer patients [33]. In light of this evidence, aneuploidy can be rapidly and precisely revealed by means of iFC, as changes in the DNA content by measuring the relative PI fluorescence intensity—as mirrored by DNA index alterations—of cancer cells compared to their normal counterparts. In our samples, 2 out of 11 cancer samples and one respective margin had a DNA index of 0.8, revealing a hypoploidy pattern that might be associated with NMSC development and progression.

The implementation of iFC offers a groundbreaking approach in the intraoperative assessment of NMSC. The precision to rapidly and accurately identify the DNA content and tumor index in real time is paramount in ensuring complete tumor excision and minimizing the risk of recurrence. As showcased in Figure 1, the combination of iFC with traditional histological examinations augments the diagnostic accuracy. The corroboration between the iFC and histological findings underscores the potential of integrating this innovative technology into current surgical practices for NMSC.

The application of iFC for NMSC presented challenges such as the heterogeneous nature of NMSC cells and the skin’s layered structure. Flow cytometry analysis of BCC, in particular, could be jeopardized by its several histological subtypes and its generally low aggressiveness [34]. In addition, unlike other cancers where tumor boundaries are more evident, NMSC often requires more nuanced analysis [35].

Moreover, the cancer cells can evade the natural apoptosis cascade by maintaining proliferative signaling, eluding growth suppressors, resisting cell death, and achieving replicative immortality [28]. Consequently, a higher proportion of cells enter the S and G2/M phases, and this increased proliferative potential is directly captured with the tumor index variable calculated using the iFC process. Conversely, we showed a notable decrease in the proportion of G0/G1 cells in all cancer samples compared to the controls, which further supports the increased proliferative potential of malignancy, and the diagnostic capability of the iFC in the detection of NMSC. The DNA index analysis revealed only two samples with hypoploidy.

Even more important is the impact of flow cytometry in the intraoperative assessment of skin cancer resection margins. Although NMSCs are often uniformly regarded as indolent cancers, this is true only for small skin tumors, treated early and adequately. In fact, these tumors are characterized by invasive growth, causing destruction of the surrounding tissues, while distant metastasis occurs rarely in BCC, compared to the higher rate of SCC [13,15,16]. The European consensus-based interdisciplinary guidelines for BCC have proposed the terms ‘easy-to-treat (common) and ‘difficult-to-treat’ BCC, while the National Comprehensive Cancer Network (NCCN) guidelines are more straightforward, using the classification of low risk and high risk for recurrence BCC, based on clinical and pathological tumor characteristics, with recurrence being a clear feature of aggressiveness [33,36]. Similarly, SCC is classified as low risk and high risk, or low, high, and very high risk for recurrence by the European and NCCN guidelines, respectively [13,15]. Overall, failure to resect a NMSC completely predisposes to recurrence, enhances the aggressiveness of the tumor, and the metastatic potential of SCC, while increasing the risk of deleterious consequences significantly if the tumor is in an aesthetically important area (such as eyelids, nose, lips and ears) [13,33,36].

Moreover, the presence of positive margins in the pathological report results in negative effects, such as additional surgery, increased risk for surgical complications and poor cosmetic outcome, additional psychological stress, delay of potential adjuvant therapies, and an additional financial burden for the health system [14]. Several techniques have been developed to reassure the surgeon that the whole tumor has been excised during the operation. Mohs micrographic surgery has been suggested as the preferred surgical treatment option for local, high-risk BCC and any risk local cutaneous SCC, because it entails the intraoperative analysis of 100% of the excision margin [13,33,36]. However, the Mohs technique is notorious for its cumbersome, time-consuming process for the specimen, with the tumor excision and repair of a single defect often to being extended for two days. In addition, certain tumor characteristics, such as BCC location (H-zone), diameter (BCC > 1.1 cm, SCC < 2 vs. ≥2 cm: 98.1% vs. 74.8%), aggressive BCC subtype or SCC differentiation (well vs. poorly differentiated: 97.0% vs. 67.4%), and recurrent tumors are associated with more Mohs stages and lower cure rates [13,33,36].

Recently, tumor excision with Peripheral and Deep En Face Margin Assessment (PDEMA), either with permanent section or intraoperative frozen section analysis, has emerged as an acceptable alternative to Mohs for NMSC [13,33,36]. Lower recurrence rates compared directly to standard excision have been reported, where histologically clear margins are achieved [37]. It is important though to note that truly histologically negative margins are not necessarily achieved for SCC using frozen section analysis alone, without PDEMA. In fact, the permanent paraffin section analysis revealed that the combined incomplete and very narrow (<1 mm) excision margin rates were 28.7% and 27.5% for BCC and SCC, respectively, attributing these rates to unrepresentative sampling of the margins [38]. Poulsen et al. studied potential risk factors associated with 12-month recurrence and non-radical NMSC excision, where the tumor has been excised with intraoperative, frozen-section histopathological assessment [39]. They showed that patients with certain BCC subtypes (superficial, morpheaform, infiltrative BCC) require frozen section, while incomplete BCC removal is more frequent on the ear [39].

Furthermore, the recent COVID-19 pandemic, and the subsequent restrictions to healthcare services, had a profound impact on skin cancer diagnosis and treatment [40,41]. Indeed, an increased incidence of SCC and more complicated methods of reconstruction following NMSC excision, as well as higher melanoma burden and disease progression were revealed [40,41]. Considering the association between delay of surgery and increased mortality recently revealed, and the heavy workload of NMSC cases anticipated in the post-pandemic era, the timely and adequate treatment provided is of utmost importance [42].

All these procedural limitations highlight the importance of a rapid, detailed, and accurate intraoperative process, such as iFC, for the NMSC margins’ assessment, providing valuable information regarding the tumor removed, enabling adequate decision making and better intraoperative management, which directly affects cure rates, and lowers the recurrence rates. This study’ results, confirmed using the gold-standard method of histopathologic evaluation performed in parallel, are very promising in the surgical management of NMSC. Cutoff values for sensitivity and specificity were defined (~95% sensitivity and ~87% specificity, increasing the accuracy of this method to ~91%, and exceeding the corresponding values of other available modalities). These outcomes are expected to be confirmed with next-generation sequence analysis, which enables the molecular characterization of tumors, thus emerging as a critical element in the therapeutic and clinical patients’ care [43,44]. Other non-invasive modalities, such as optical coherence tomography, reflectance confocal microscopy, and deep Raman spectroscopy, have recently emerged in the field of dermatooncology, aiming mainly to improve the diagnostic capability and accuracy regarding skin tumors [45,46,47]. Major drawbacks remains though, including the limited information provided regarding tumor depth, and thus complete margin assessment.

Certain limitations apply to this study, namely the restricted scope of the analysis to NMSC, the small number of analyzed samples, and the reliance on data from a single institution. Our main objective was to provide preliminary data on the potential use of intraoperative flow cytometry (iFC) in diagnosing NMSC and its surgical clearance. Given the promising results of this pilot study, our aim is to continue into a larger study to further validate our findings. The ability of our assay to differentiate tumor from normal tissue, and the experience accumulated with the technique and this specific tissue, indicates that IFC has potential for an even higher sensitivity and specificity rate. However, such an assumption needs to be verified. Multicenter studies will enable a greater skin lesion qualitative and quantitative analysis, leading to our results’ verification, while showing other procedural approaches. Certainly, the iFC DNA study is a one-dimensional analysis. While the precision of iFC, grounded in DNA-content measurement, has been demonstrated across various cancers, a more detailed characterization of skin cancer cells might be achieved through immunophenotypic flow cytometric evaluation, an interesting future perception that needs to be further assessed for its speed and accuracy [48,49]. The evolution of such an assessment, focusing on immunophenotyping beyond just DNA study, is anticipated to provide fresh perspectives on the diagnostic, prognostic, and therapeutic significance of iFC.

As we extrapolate these findings, the potential implications for improving surgical precision, reducing recurrence rates, and enhancing patients’ postoperative quality of life become apparent. Future studies aiming at the optimization of iFC parameters and protocols, and expanding the patient cohort, will be instrumental in establishing this technique as a standard component in NMSC surgical procedures.

## 5. Conclusions

Overall, intraoperative DNA content and cell-cycle evaluation using flow cytometry offer dependable data for the precise pinpointing of NMSC lesions, as well as gauging its severity and providing thorough margin removal. As a result, this innovative method might serve as an adjunct, supplementary tool to the conventional pathology assessment in the skin cancer domain, facilitating a credible and precise description of the NMSC and its surgical margins. Validation of our results with next-generation sequencing and further multicenter studies will provide the ground for wider application of iFC towards the goal of individualized and precise cancer medicine.

## Figures and Tables

**Table 1:** Clinical, pathological, and intraoperative flow cytometry data.

Patient	Age (y)	Site	Tumor Type	Size (mm)	Excision Margins (mm)		DNA Index	Tumor Index
					Peripheral	Deep		
A.L.	89	Forehead	BCC	7	3	2.1	1	28
A.L.	89	Nose	BCC	8	2.3	1	1	13
S.G.	89	Neck	BCC	9	1.8	2.5	1	7
O.S.	43	Nose	BCC	15	-	0.2	0.8	10
S.G.	63	Ear	SCC	27	3	0.7	1	6
K.A.	86	Forehead	SCC	5	2	2	0.8	9
A.B.	85	Cheek	BCC	25	1	0.8	1	18
P.P.	91	Nose	BCC	11	2	2.3	1	37
G.E.	78	Temple	BCC	5	1.5	2	1	12
P.M.	83	Dorsum	AK	15	4	3	1	5
D.A.	90	Cheek	BCC	10	0.2	-	1	9
F.G.	89	Temple	SCC	46	-	-	1	8
K.A	88	Forehead	SCC	21	4	1.2	1	16
D.C.	70	Nose	BCC	13	1	1.5	1	7
K.G.	75	Medial Canthus	BCC	3	3	0.5	1	6
P.C	64	Scalp	SCC	26	1.5	-	1	10
V.A.	86	Cheek	BCC	21	2	0.2	1	13
M.G.	72	Cheek	BCC	3	4	4	1	11
G.T.	84	Postauricular	BCC	15	-	-	1	20
B.A.	79	Hand	SCC	15	4	2	1	35
D.R.	91	Temple	BCC	30	-	0.5	1	35
S.D.	84	Upper Lip	BCC	11	0.8	4	1	8
S.V.	64	Cheek	SCC	16	4	3	1	7
N.K.	75	Nose	BCC	8	1	1	1	7
L.P	73	Nose	AΚ	10	-	-	1	6
L.P	73	Forehead	BCC	7	1	1	1	15
I.G	58	Ear	BCC	7	-	0.5	1	6
G.E.	85	Cheek	SCC	14	3	1	1	6
K.G.	62	Ear	BCC	20	0.5	0.8	1	5
M.O.	77	Forehead	SCC	26	2	2	1	23

-: Positive margin.

## Data Availability

The data presented in this study are available upon reasonable request to the corresponding author.

## Supplementary Materials

The following supporting information can be downloaded at: https://www.mdpi.com/article/10.3390/cancers16040682/s1, Figure S1. Doublet Exclusion Plot Based on Propidium Iodide Fluorescence. Table S1: Descriptive statistics for G0/G1, tumor index and DNA index, cell percentage in control vs. cancer cell populations. Table S2: ROC analysis results for discrimination between normal and cancer cells, based on G0/G1 values and determination of optimal cutoff value.
