# Supplementary material for: Intraoperative Flow Cytometry for the Rapid Diagnosis and Validation of Surgical Clearance of Non-Melanoma Skin Cancer: A Prospective Clinical Feasibility Study
Source: Cancers (Basel). 2024 Feb 6;16(4):682. doi: 10.3390/cancers16040682 (PMC10887295; doi:10.3390/cancers16040682)
Supplement: Supplementary file 1 [file cancers-16-00682-s001.zip › cancers-2833744-supplementary.pdf]

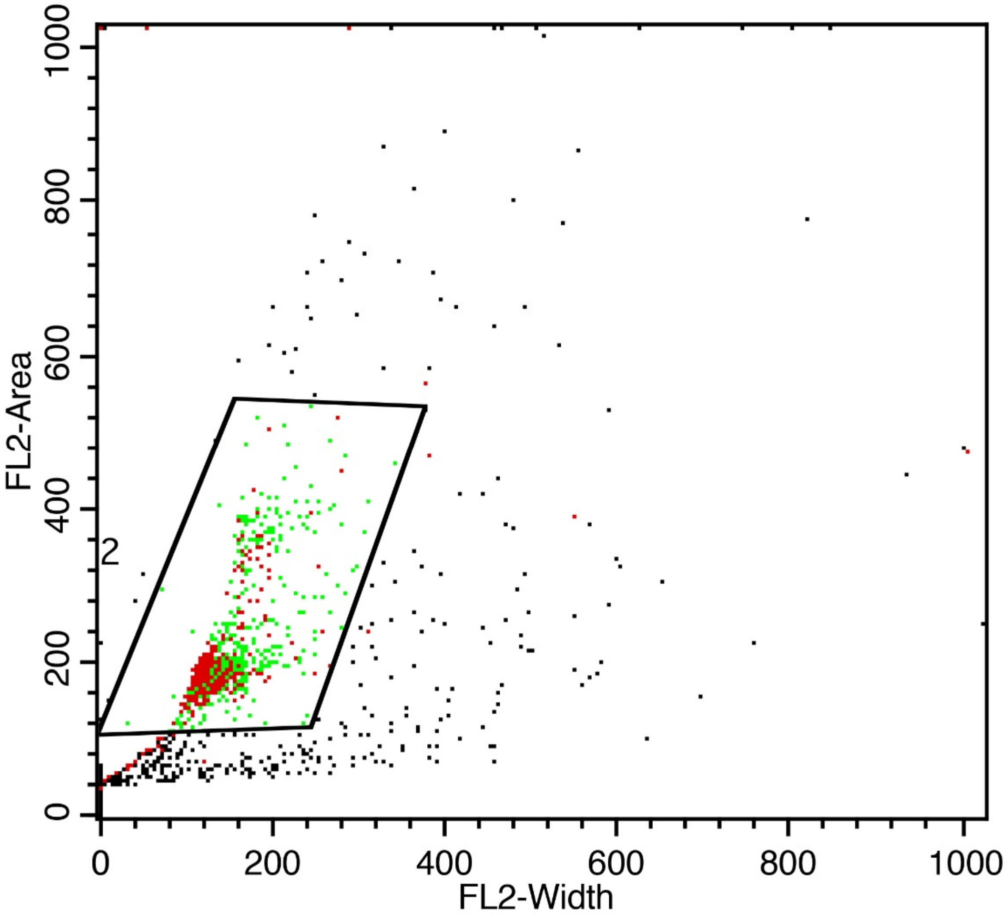

Figure S1. Doublet Exclusion Plot Based on Propidium Iodide Fluorescence: Doublet exclusion strategy employed in the study, utilizing the area and width of propidium iodide (PI) fluorescence. Individual cells are represented as points on the scatter plot, with the X-axis representing the width of PI fluorescence and the Y-axis denoting the area of PI fluorescence. Doublets or cell aggregates result in increased fluorescence area and width compared to individual cells. The delineation between single cells and doublets is clearly visible in the presented rectangular gate, enabling the exclusion of doublets to ensure the accuracy of subsequent flow cytometric analyses. This doublet exclusion is instrumental in enhancing the precision of cellular characterization, particularly critical in assessing the proliferative potential of tumor and margin samples by iFC during NMSC surgeries.

Table S1: Results of iFC analysis in NMSC surgery

DNA index	G0/G1	S	G2/M	TUMOR index	Sample type (C=Cancer, M=Margin)
1	72	3	25	28	C
1	93	2	5	7	C
0,8	90	4	6	10	C
1	94	2	4	6	C
0,8	91	6	3	9	C
1	82	8	10	18	C
1	63	4	33	37	C
1	88	5	7	12	C
1	95	2,5	2,5	5	C
1	91	5	4	9	C
1	92	4	4	8	C
1	84	12	4	16	C
1	93	5	2	7	C
1	94	4	2	6	C
1	90	9	1	10	C
1	87	9	4	13	C
1	88	9	2	11	C
1	80	10	10	20	C
1	65	10	25	35	C
1	65	31	4	35	C
1	92	5	3	8	C
1	93	5	2	7	C
1	93	6	1	7	C
1	85	12	3	15	C
1	94	5	1	6	C
1	94	5	1	6	C
1	94	4	2	6	C
1	95	4	1	5	C
1	77	8	15	23	C
1	91	2	7	9	M
1	97	1	2	3	M
1	87	7	6	13	M
1	93	3	4	7	M
1	90	4	6	10	M
1	94	2	4	6	M
1	98	1	1	2	M
1	95	2	3	5	M
1	95	2	3	5	M
0,8	93	4	3	7	M
1	97	2	1	3	M

1	90	7	3	10	M
1	92	2	6	8	M
1	94	2	4	6	M
1	90	5	5	10	M
1	95	2	3	5	M
1	96	2	2	4	M
1	97	1	2	3	M
1	96	2	2	4	M
1	95	3	2	5	M
1	94	3	3	6	M
1	95	3	2	5	M
1	91	5	4	9	M
1	98	1	1	2	M
1	96	2	2	4	M
1	94	4	2	6	M
1	93	5	2	7	M
1	97	1	2	3	M
1	97	1	2	3	M
1	96	3	1	4	M
1	92	7	1	8	M
1	93	4	3	7	M
1	96	3	1	4	M
1	90	7	3	10	M
1	96	2	2	2	M
1	95	3	2	5	M
1	83	9	8	17	M
1	96	2	2	4	M
1	95	4	1	5	M
1	93	5	2	7	M
1	95,5	2,5	2	4,5	M
1	96	2	2	4	M
1	96	2	2	4	M
1	95	3	1	4	M
1	96	3	1	4	M
1	98	1	1	2	M
1	98	1,5	0,5	2	M
1	99	1	0	1	M
1	99	1	0	1	M
1	99	1	0	1	M
1	98	1	1	2	M
1	97	2	1	3	M
1	93	6	1	7	M
1	98	1,5	0,5	2	M
1	99	1	0	1	M
1	97	2	1	3	M
1	97	2	1	3	M
1	98	1	1	2	M
1	97	1	2	3	M

Table S2: ROC Curve analysis results.

Coordinates of the Curve

Test Result Variable(s): TUMOR index

Positive if Greater Than or Equal To[a]	Sensitivity	1 – Specificity
,000	1,000	1,000
1,500	,977	,932
2,500	,977	,750
3,500	,977	,545
4,250	,977	,318
4,750	,977	,295
**5,500**	**,909**	**,159**
6,500	,773	,091
7,500	,568	,068
8,500	,500	,045
9,500	,409	,045
10,500	,318	,000
11,500	,295	,000
12,500	,273	,000
14,000	,227	,000
15,500	,205	,000
16,500	,182	,000
17,500	,159	,000
19,000	,136	,000
21,500	,114	,000
25,500	,091	,000
31,500	,068	,000
36,000	,023	,000
38,000	,000	,000

a. The smallest cutoff value is the minimum observed test value minus 1, and the largest cutoff value is the maximum observed test value plus 1. All the other cutoff values are the averages of two consecutive ordered observed test values.
